# Supplementary material for: Go Get Data (GGD) is a framework that facilitates reproducible access to genomic data
Source: Nat Commun. 2021 Apr 12;12:2151. doi: 10.1038/s41467-021-22381-z (PMC8041854; doi:10.1038/s41467-021-22381-z)
Supplement: Supplementary file 1 — Supplementary Information [file 41467_2021_22381_MOESM1_ESM.pdf]

Supplemental Note

GGD Continuous Integration System:
The GGD continuous integration (CI) system is an automated workflow for: running unit and functional tests for the GGD command-line interface (CLI), testing new recipes, packaging recipes, uploading data to caching services (currently AWS S3), integrating recipes into GGD specific Conda channels, maintaining global level metadata used by GGD, documentation updates, and the relevant testing and maintenance of the GGD ecosystem.

In-depth unit and functional tests for the GGD CLI have been developed to ensure GGD works properly in POSIX systems. The GGD CI system will run each unit and functional test for each GGD-supported Python version on each GGD-supported platform every night. This ensures that the GGD CLI works correctly and that any potential breaking changes in GGD or from dependencies would be found and fixed promptly. The CI system will run all GGD unit and functional tests on all GGD supported versions of Python and platforms. It is adaptable to changes or additions to the Python version and platforms as the system evolves.

The GGD CI system has an extensive testing and validation schema for new or updated recipes that are added to the GGD ecosystem. Multiple levels of testing are done to ensure that the recipe will work with Conda and GGD on different platforms.

First and foremost, because GGD is built on Conda, a new or updated GGD recipe must be compliant with the Conda system. The CI system will check each recipe’s compliance with Conda structure and standards to ensure it is a proper Conda recipe. This ensures that it can be used and recognized by the Conda system. Using Conda, the CI system will also check that the recipe can be packaged into a standard Conda package and test its installation.

It is common for a GGD recipe to have software and data dependencies. The CI system will check that software dependencies are available through Conda and that data dependencies are available through GGD. The CI system will further test that these dependencies are properly installed during a recipe's installation. Furthermore, the CI system will check that the dependency list supplied within the recipe is sufficient and that no other dependencies are needed to install the recipe.

The CI system will check the recipe contents and structure to ensure that all necessary pieces of a GGD recipe are provided. The system will also check that the steps outlined in the recipe are processed without error. Recipe installation with GGD is checked. The list of final data files installed by the recipe is validated, along with removing any intermediate data files. The file type of the final data files is checked, and the md5sum checksum hash values are validated. Each final data file’s installation path is checked and validated to ensure that it exists in its proper location.

Furthermore, the CI system is set up to test specific data file types. The list of checked file types is easily adapted so that many more file types can be added. The tests that are done on

different file types depend on the file type itself. For example, VCF files are checked to ensure that they have a proper header, including information on contig length, info fields, formatting fields, etc., along with checking that variant entries are formatted correctly. Files with genomic positions such as GTF, BED, etc., are checked for a proper header and proper formatting. Genomic position-based files such as VCF, GTF, BED, etc., are checked for appropriate chromosome labeling, sorting, compression and indexing, etc. Alignment files like SAM and BAM files are checked for proper headers, formatting, and info based on current standards. Other files, such as txt, csv, etc. are checked for an associated header for each field in the file. Adding tests to the CI system based on different file types is easy, making it adaptable to a wide range of possible data files as they become available.

The CI system runs all tests and checks for new/updated GGD data recipes on all supported GGD platforms. This ensures that the recipe will work across all available platforms regardless of platform structure and software differences, mitigating the need for platform-specific data recipes.

If a recipe passes all tests and validation, the CI system will package the recipe and add it to a GGD specific Conda channel. For recipes set to be cached, the CI system will update the recipe for caching, run through the testing and validation steps, add the updated cache specific recipe to a GGD specific Conda channel, and upload the final data files to the caching source. The CI system will check and update all appropriate global metadata if a new/updated recipe is added to a GGD specific Conda channel.

The GGD data recipe CI system is run every night to check for new/updated recipes that need to be tested. Additionally, each night the CI system will check GGD metadata and update as needed.
