# Supplementary material for: Go Get Data (GGD) is a framework that facilitates reproducible access to genomic data
Source: Nat Commun. 2021 Apr 12;12:2151. doi: 10.1038/s41467-021-22381-z (PMC8041854; doi:10.1038/s41467-021-22381-z)
Supplement: Supplementary file 3 — Reporting Summary [file 41467_2021_22381_MOESM3_ESM.pdf]

nature research

Corresponding author(s): Aaron Quinlan

Last updated by author(s): YYYY-MM-DD

Reporting Summary

Nature Research wishes to improve the reproducibility of the work that we publish. This form provides structure for consistency and transparency in reporting. For further information on Nature Research policies, see our Editorial Policies and the Editorial Policy Checklist.

Statistics

For all statistical analyses, confirm that the following items are present in the figure legend, table legend, main text, or Methods section.

n/a	Confirmed	
☒	☐	The exact sample size (*n*) for each experimental group/condition, given as a discrete number and unit of measurement
☒	☐	A statement on whether measurements were taken from distinct samples or whether the same sample was measured repeatedly
☒	☐	The statistical test(s) used AND whether they are one- or two-sided<br>*Only common tests should be described solely by name; describe more complex techniques in the Methods section.*
☒	☐	A description of all covariates tested
☒	☐	A description of any assumptions or corrections, such as tests of normality and adjustment for multiple comparisons
☒	☐	A full description of the statistical parameters including central tendency (e.g. means) or other basic estimates (e.g. regression coefficient) AND variation (e.g. standard deviation) or associated estimates of uncertainty (e.g. confidence intervals)
☒	☐	For null hypothesis testing, the test statistic (e.g. *F*, *t*, *r*) with confidence intervals, effect sizes, degrees of freedom and *P* value noted<br>*Give P values as exact values whenever suitable.*
☒	☐	For Bayesian analysis, information on the choice of priors and Markov chain Monte Carlo settings
☒	☐	For hierarchical and complex designs, identification of the appropriate level for tests and full reporting of outcomes
☒	☐	Estimates of effect sizes (e.g. Cohen's *d*, Pearson's *r*), indicating how they were calculated

Our web collection on statistics for biologists contains articles on many of the points above.

Software and code

Policy information about availability of computer code

Data collection	No data was collected/used in the manuscript. GGD describes a data management tool that can be used for genomic data access. Information on data available through GGD can be found at the gogetdata/ggd-recipes GitHub repo: https://github.com/gogetdata/ggd-recipes and on the GGD docs page: https://gogetdata.github.io/
Data analysis	No data analysis was performed in this manuscript.<br>All code associated with GGD can be found at https://github.com/gogetdata.<br>GGD is built in Python and requires Python version >= 3.6<br>GGD recipes are made up of Bash scripts using version >= 3.2<br>GGD utils are built in GO using version 1.13<br>GGD documentations are built with Sphinx version 3.4 and are hosted as a combination of html, CSS, and JavaScript files.<br>GGD metadata are represented as json files

For manuscripts utilizing custom algorithms or software that are central to the research but not yet described in published literature, software must be made available to editors and reviewers. We strongly encourage code deposition in a community repository (e.g. GitHub). See the Nature Research guidelines for submitting code & software for further information.

Data

Policy information about availability of data

All manuscripts must include a data availability statement. This statement should provide the following information, where applicable:

- Accession codes, unique identifiers, or web links for publicly available datasets
- A list of figures that have associated raw data
- A description of any restrictions on data availability

GGD is a data management system developed to help provide sustainable, accurate, reproducible data. No data was used in this manuscript, but data hosted by GGD is available at: https://github.com/gogetdata/ggd-recipes and through the GGD command line interface: https://github.com/gogetdata/ggd-cli. Additional information can be found on the documentation page at: https://gogetdata.github.io/.

Field-specific reporting

Please select the one below that is the best fit for your research. If you are not sure, read the appropriate sections before making your selection.

[x] Life sciences [ ] Behavioural & social sciences [ ] Ecological, evolutionary & environmental sciences

For a reference copy of the document with all sections, see nature.com/documents/nr-reporting-summary-flat.pdf

Life sciences study design

All studies must disclose on these points even when the disclosure is negative.

Sample size	No sampling was performed in this work.
Data exclusions	There is no data exclusion.
Replication	This work does not include any experiments. Replication does not apply.
Randomization	This work does not include any experiments. No randomization was done.
Blinding	This work does not include any experiments. No blinded study was done.

Reporting for specific materials, systems and methods

We require information from authors about some types of materials, experimental systems and methods used in many studies. Here, indicate whether each material, system or method listed is relevant to your study. If you are not sure if a list item applies to your research, read the appropriate section before selecting a response.

Materials & experimental systems

n/a	Involved in the study	
[x]	[ ]	Antibodies
[x]	[ ]	Eukaryotic cell lines
[x]	[ ]	Palaeontology and archaeology
[x]	[ ]	Animals and other organisms
[x]	[ ]	Human research participants
[x]	[ ]	Clinical data
[x]	[ ]	Dual use research of concern

Methods

n/a	Involved in the study	
[x]	[ ]	ChIP-seq
[x]	[ ]	Flow cytometry
[x]	[ ]	MRI-based neuroimaging
